# Brain network functional connectivity changes in long illness duration chronic schizophrenia

**DOI:** 10.3389/fpsyt.2024.1423008

**Published:** 2024-06-19

**Authors:** Yin Xia Bai, Jia Xin Luo, Duo Peng, Jing Jing Sun, Yi Fang Gao, Li Xia Hao, B. G. Tong, Xue Mei He, Jia Yu Luo, Zi Hong Liang, Fan Yang

**Affiliations:** ^1^ Department of Psychiatry, Inner Mongolia Mental Health Center, Hohhot, China; ^2^ Department of Psychiatry, Inner Mongolia Brain Hospital, Hohhot, China; ^3^ Department of Psychiatry, Inner Mongolia People’s Hospital, Hohhot, China; ^4^ Department of Research, Inner Mongolia Academy of Medical Science, Hohhot, China; ^5^ Department of Rehabilitation, Genghis Khan Community Branch of Inner Mongolia People’s Hospital, Hohhot, China

**Keywords:** functional magnetic resonance imaging, independent component analysis, brain functional connectivity, schizophrenia, chronic schizophrenia

## Abstract

**Introduction:**

Chronic schizophrenia has a course of 5 years or more and has a widespread abnormalities in brain functional connectivity. This study aimed to find characteristic functional and structural changes in a long illness duration chronic schizophrenia (10 years or more).

**Methods:**

Thirty-six patients with a long illness duration chronic schizophrenia and 38 healthy controls were analyzed by independent component analysis of brain network functional connectivity. Correlation analysis with clinical duration was performed on six resting state networks: auditory network, default mode network, dorsal attention network, fronto-parietal network, somatomotor network, and visual network.

**Results:**

The differences in the resting state network between the two groups revealed that patients exhibited enhanced inter-network connections between default mode network and multiple brain networks, while the inter-network connections between somatomotor network, default mode network and visual network were reduced. In patients, functional connectivity of Cuneus_L was negatively correlated with illness duration. Furthermore, receiver operating characteristic curve of functional connectivity showed that changes in Thalamus_L, Rectus_L, Frontal_Mid_R, and Cerebelum_9_L may indicate a longer illness duration chronic schizophrenia.

**Discussion:**

In our study, we also confirmed that the course of disease is significantly associated with specific brain regions, and the changes in specific brain regions may indicate that chronic schizophrenia has a course of 10 years or more.

## 1 Introduction

Schizophrenia, a chronic mental disorder that generally emerges in young adulthood, is regarded as the extreme end of a continuum of psychotic symptoms (1). Typical symptoms of schizophrenia are grouped into three categories: distortion of reality (delusions and hallucinations); confusion (formal thinking disorder, chaotic behavior, inappropriate rare symptoms); and negative symptoms or so-called clinical poverty syndrome. In recent years, cognitive impairment has also been accepted as another clinical feature of schizophrenia (2, 3). Patients with chronic schizophrenia have a long course of disease, and in the course of treatment, about half of the patients suffer varying degrees of impact on themselves—which extends to their families and society—because of recurring episodes or their unstable mental state (1). Therefore, psychiatrists are urgently seeking breakthroughs at the levels of genes, -omics, brain functional structure, and behavior to identify biomarkers and targeted therapies for schizophrenia, with the ultimate aim of improving patients’ physical-mental-psychological state and facilitating their return to society.

Functional magnetic resonance imaging (fMRI) enables visualization and analysis of the pathophysiological changes that occur in neuro-psychiatric diseases (4). fMRI can therefore be used to observe the neural mechanisms involved in such diseases based on the spontaneous activity indicators of brain regions (such as local consistency, low-frequency amplitude fraction) as well as the synergistic indicators between brain regions, such as functional connectivity (FC) (5). The human brain involves a complex network of functional connections (6). Previous brain imaging studies have divided brain networks into two major categories: the somatic sensory network:visual network (VIN), auditory network (AUN), and somatomotor network (SMN),and high-level cognitive network: default mode network (DMN), fronto-parietal network (FPN), and dorsal attention network (DAN) based on FC strength of the brain’s low-frequency signals combined with the brain regions (7, 8). Functional brain networks identified from fMRI data may serve as a source of potential biomarkers for numerous mental disorders (9).

Independent component analysis (ICA) is one of the most widely applied multivariate methods for analyzing brain functional networks (10). ICA enables the simultaneous analysis of multiple brain networks by considering the whole fMRI data, and can also denoise the fMRI data by decomposing artifacts as independent components (ICs), thereby extracting more meaningful components (11, 12). The ICA method is used to study the strength of functional connections within and between brain networks, identify the various types of signals according to their spatial and temporal characteristics—dependent on the blood oxygen level of the brain, and extract the brain network signals in the resting state of the brain, with high sensitivity and reproducibility. In recent years, ICA technology has been widely used in the study of neurodegeneration (13). Patients with chronic schizophrenia tend to have a long disease course, experience significant degenerative brain changes, and exhibit mental disorders (1).

Meda et al. reported abnormal functional network connectivity (FNC) in patients with schizophrenia and psychotic bipolar disorder (14). Studies have found that patients with schizophrenia exhibit activation disorders or FC abnormalities in multiple brain regions—i.e., the “disconnection hypothesis”—that are associated with cognitive deficits (2). Temporally coherent brain networks, such as temporal lobe and DMN, have been shown to reliably discriminate between subjects with bipolar disorder, patients with chronic schizophrenia, and healthy individuals (15). Recent studies have shown that in patients with schizophrenia, changes in the brain FC occur; psychotherapy can alleviate these changes to a certain extent, and the effect of treatment is directly reflected in the imaging results (16). Gallos er al. reported differences in DMN and FPN for schizophrenia and suggest functional networks as biomarkers for the monitoring of the disease in the course of psychotherapy, they also use ICA for identifying these networks (17).Changes in brain function occur in different subregions of the frontal cortex and may ultimately be understandable in terms of disturbed interactions between brain networks; in this regard, multimodal studies based on brain networks suggesting that abnormal basal ganglia-thalamic-cortical circuits can serve as biomarkers for schizophrenia (1, 18). Although ICA methods have identified potential biomarkers for schizophrenia, to date, there are no studies that systematically analyze the correlation of specific biomarkers in and between brain networks with clinical data in patients with chronic schizophrenia.

With the development of medical level, in recent years, the treatment environment of schizophrenia patients has been significantly improved than in the past, and there are fewer and fewer patients with long disease course and multiple attacks. Most of the patients in our study had a disease course of more than 10 years, and all had multiple episodes, which was relatively rare in the existing studies. Therefore, this study aimed to find characteristic functional and structural changes and the indicators of brain network connectivity in a long illness duration chronic schizophrenia (10 years or more) using ICA technology.

## 2 Materials and methods

**Figure 1:**
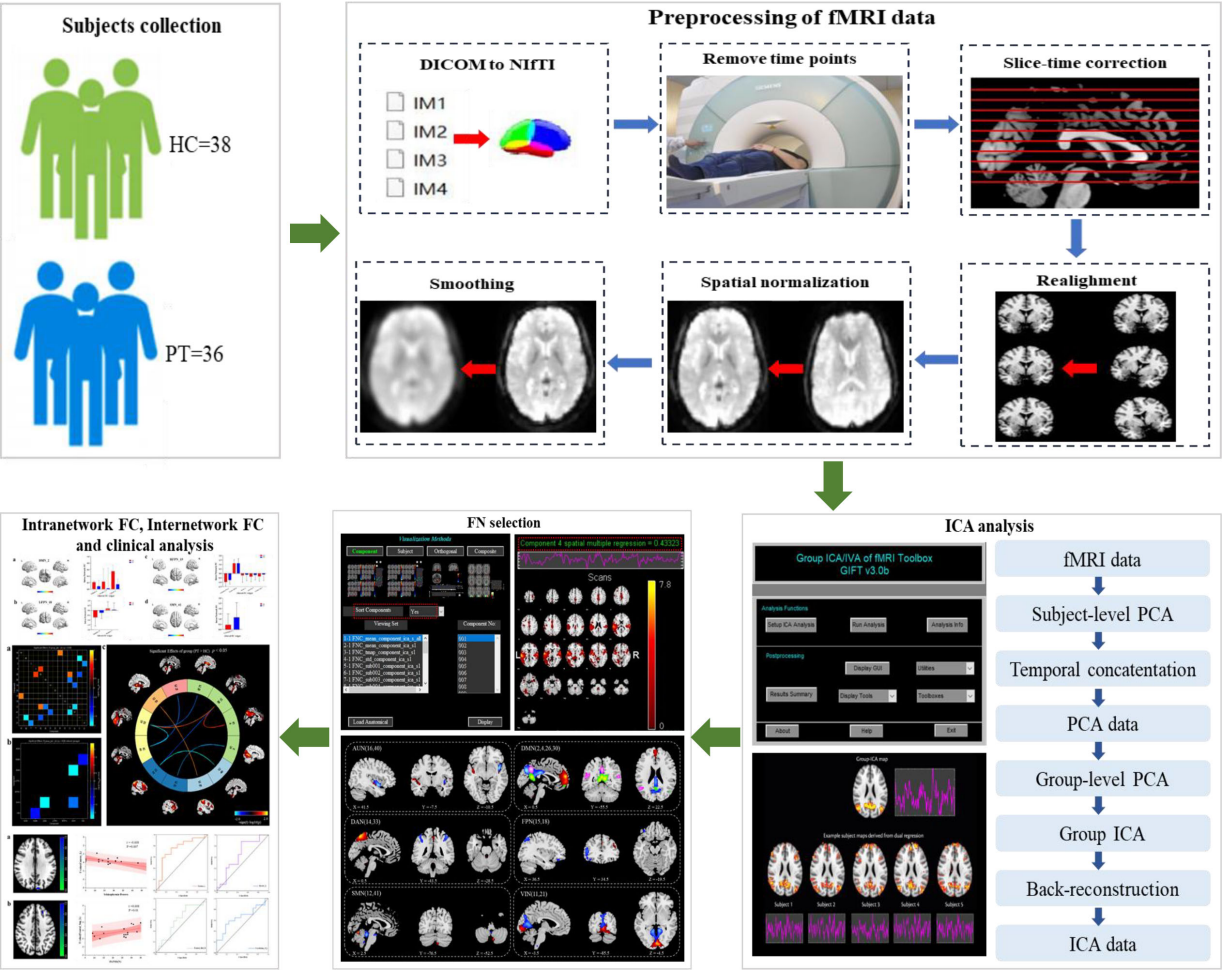
Diagram of the processing flow. Step one: subjects collection. Step two: preprocessing of fMRI data. Step three: ICA analysis. Step four: FN selection. Step five: Intranetwork FC, internetwork FC and clinical analysis.

This study aimed to investigate the characteristics of functional brain networks in patients with chronic schizophrenia and their relationship with clinical symptoms using ICA (**Figure 1**)

### 2.1 Subjects

A cohort of 40 patients with chronic schizophrenia as Patient group (PT) and 40 healthy peoples as Control group (HC) were recruited from the Inner Mongolia Mental Health Center between June 2019 and December 2022. All subjects were right-handed.

Patients met the following inclusion criteria (19): 1) All patients met the diagnostic criteria of the Structured Clinical Interview for Diagnostic and Statistical Manual (DSM)-IV-TR axis 1 disorders(SCID-I-P); 2) patients were aged between 20 and 60 years; 3) patients’ duration of illness was at least five years; 4) patients had experienced at least two psychotic episodes; 5) patients had a minimum score of 60 on PANSS (20). Exclusion criteria were as follows: 1) history of major chronic medical or neurological conditions; 2) past or current significant drug/alcohol abuse, other than nicotine. HC were interviewed to confirm a lifetime absence of psychiatric illnesses and to exclude a known history of psychiatric illness in first-degree relatives.

All the subjects were of Chinese Han descent. There was no biological relationship between PT and HC. Age, sex, and years of education were approximately matched between PT and HC. All subjects provided written informed consent for participation in the research. The study was approved by the Ethics Committee of Mental Health Center of Inner Mongolia Autonomous Region (Ethical code: [2024] Ethics Review No. 27) in March 17, 2024.

### 2.2 Measures

Psychotic symptoms were assessed based on PANSS (20). The primary outcomes were the total PANSS score (PANSS-T), positive score (PANSS-P), negative score (PANSS-N), and general symptoms score (PANSS-G). Beside these scores, three factors from the five-factor model of PANSS—namely, the cognitive factor, excitement factor, and anxiety/depression factor—were also evaluated and analyzed (21).

Cognitive function was evaluated based on the Wisconsin Card Sorting Test (WCST) (22); in this study, a computer-based version by RiRiXin software Inc (1.0 version) was used, with the 128 cards varying according to color (red, yellow, blue, green), shape (triangle, cross, circle, pentagon), and number (1, 2, 3, 4). Assessment indicators include the number of categories achieved, total errors, perseverative errors, non-perseverative errors, and conceptual level response.

### 2.3 fMRI data collection and preprocessing

#### 2.3.1 fMRI data acquisition

All imaging was performed using a 3.0-Tesla Magnetic resonance system (Achieva TX, Phillips, Netherlands). High-resolution T1-weighted images were obtained using a volumetric 3D spoiled gradient recall sequence (TR=7.4 ms, TE=3.4 ms, flip angle=8, slice thickness=4.0 mm, resolution=228 × 228 matrix, FOV=25 cm, 230 slices) and a 16-channel phased-array head coil. Total acquisition time was 6 min 53 seconds. The resting-state echo-planar imaging scan (TR=2200 ms, TE=35 ms, 50 axial slices, slice thickness=3 mm, slice gap=1 mm, voxel size=3.4 × 3.4 × 3 mm^3^, flip angle=90^°^, FOV=22 × 22 cm^2^, resolution=96 × 96 matrix) lasted 17 min 40 seconds. All subjects were asked to rest quietly with their eyes closed, lie still, relax, and avoid falling asleep during the scanning period.

#### 2.3.2 fMRI data preprocessing

The Matlab2018 platform was used to preprocess the MRI data using the auxiliary tool software RESTplus 1.2 based on the SPM12 software system (23, 24). The specific preprocessing steps were as follows:

1) Conversion of format, which involves conversion of the original DICOM-format fMRI data file into a NIFTI format that can be recognized by the software;
2) Removal of first time points: because the magnetic field is unstable at the beginning of the MRI scan, it takes time for the magnetic field to reach a steady state; furthermore, the individual undergoing the scan is often nervous due to the unfamiliar environment, which makes the scan data from the beginning of the scan less reliable. Therefore, data for the first 10 time points were excluded in the first step of the MRI pre-data preprocessing;
3) Slice Timing: when scanning the brain, there are generally a variety of different scanning methods, such as scanning layers 1, 3, 5, 7, and 9 first, and then scanning layers 2, 4, 6, 8, and 10; therefore, the first and third layers are scanned relatively early, while the second layer is scanned relatively late. Therefore, the scanning time for the same brain region may be quite different, and it is necessary to apply mathematical methods to correct them to the same time point to scan to eliminate the difference;
4) Head movement correction (Realign): The subjects will inevitably move their heads during the scanning process, and any slight movement of the head will cause the position of the activated voxel to move, thereby changing the real functional signal. Therefore, specific algorithms are necessary for head motion correction, including translation and rotation. In this trial, data from subjects with a head movement range of more than 3 mm and a rotation angle of more than 3° were excluded from analysis.
5) There are individual differences in spatial standard, head circumference, and brain structure. To reduce the impact of this difference on the research results, it is necessary to standardize the average functional images of all subjects’ heads to the same space before they can be compared with each other. Spatial standardization was performed to segment the corrected images with a standard of 3 mm × 3 mm × 3 mm, which were then registered to the standard spatial template of the Montreal Institute of Neurology (25).
6) Smoothing: The advantage of smoothing is that it can reduce the inaccuracy of registration and improve the signal-to-noise ratio. The Gaussian kernel function of 6 mm full width and half height is used to smooth the data in order to enhance the normality of the data and facilitate statistical analysis.
7) High pass filter: The data of the bold signal in the frequency range of 0.01–0.08 Hz is filtered out.

### 2.4 Group-level ICA brain network analysis

We used the GIFT software toolkit (http://icatb.sourceforge.net/, version 3.0b) to calculate the number of ICs at the group level (11): 1) the software estimates the number of ICs and calculates the spatial correlation of the BOLD signal by the minimum description length criterion (26); 2) the IC data of each participant was further reduced; then, ICA was performed by using the Infomax algorithm to further calculate the number of ICs (27); 3) reverse reconstruction of the ICs of each participant was performed through group-level ICA; 4) among the final ICs, the spatial correlation of specific resting state networks (RSNs) (28) templates was selected for further analysis. We screened the brain network components according to the following criteria (29): (a) the spatial coordinate location of the brain network peak was in the gray matter of the brain; (b) distribution of brain networks: there are no obvious blood vessels in the brain area, and the suspected artifacts overlap; (c) brain network signals are mainly low-frequency signals (spectral range from 0.1 Hz to 0.15–0.25 Hz). After rigorous screening, six RSNs were identified in this study: AUN, DMN, DAN, FPN, left fronto-parietal network (LFPN) and right fronto-parietal network (RFPN), SMN, and VIN. Finally, the screening results were analyzed within and between groups. The differential values for the brain regions were increased with the xjView 10.0 toolkit and RESTplus 1.2 software, and visualized using MRIcorn_GL 1.2, prism 9.0, and Origin 2023 software.

### 2.5 Group-level ICA was performed between groups

Single-sample t-test was used to count the spatial distribution of brain networks in the two groups (P<0.001, FDR corrected). The two-sample t-test was used to compare the differences in FC within the brain network between the two groups. Multiple comparison corrections were performed. The Gaussian random field method (two-tailed, voxel level P<0.01, cluster level P<0.05) was used to compare the differences between the two groups of participants, and age and gender were used as regression covariates. In addition, the marks generated by the union of the single-sample t-test brain networks of the two groups were applied to the two-sample t-statistical analysis.

FNC was used to analyze the FC between networks, and the one-sample t-test was used to compare the temporal correlation of brain networks between each group (P<0.05). The two-sample t-test was used to compare the differences in brain network connectivity between the two groups. The FNC Toolkit was used (http://trendscenter.org/software/, version 2.3).

### 2.6 Correlation analysis between FC within the ICA network and clinical data

The differential brain region was regarded as the region of interest (ROI), and the Extract ROI Signals function in REST plus software was used to extract the intra-network FC value corresponding to the ROI of each subject. Pearson correlation analysis was performed with SPSS 27.0 statistical software to analyze the FC value corresponding to the ROI. The clinical evaluation index, and age and gender, were removed as covariates. P<0.05 was considered to indicate a correlation; the r-value represented the degree of correlation and a scatter plot was plotted using the Origin software. Receiver operating characteristic (ROC) curve of the ROI between the two groups was plotted, and the sensitivity, specificity, and area under the ROC curve were calculated to explore the optimal cut-off value of the ROI in patients with chronic schizophrenia.

## 3 Results

### 3.1 Socio-demographic data and analysis of clinical symptom scores of PT and HC

Data from six subjects (four patients and two controls) were excluded because of excessive head movement. The remaining 74 subjects (PT=36, HC=38) were included in the analysis. Among the 36 patients, 21 patients were taking one atypical antipsychotic (Olanzapine or Clozapine), 10 patients were taking two atypical antipsychotics (Olanzapine and Clozapine), and five patients taking one typical (Haloperidol) and one atypical antipsychotic (Olanzapine). The mean medication dosage converted to chlorpromazine equivalents was 322 ± 202 mg.

There were no significant differences between the groups in terms of the socio-demographic variables of gender, age, and education level (P>0.05). For the number of completed categories, the percentage of conceptual level responses in the WCST scores of PT was significantly lower than that of HC (P<0.05). In terms of the total errors, perseverative errors, non-perseverative errors, PT was significantly higher than HC (P<0.05). The demographic data and clinical symptom scores of the final sample are listed in **Table 1**.

**Table 1:** Socio-demographic and clinical characteristics of subjects.

Characteristics	PTs(n=36)	HCs(N=38)	t/*X^2^*	*p*
**Age,y**	38.69 ± 11.24	41.62 ± 5.97	0.53	**0.71**
**Gender (F/M)**	27/9	30/8	2.46	0.93
**Education,y**	11.05 ± 4.53	9.72 ± 3.06	−0.71	0.52
**Age of onset of illness**	23.52 ± 6.91	NA	NA	NA
**Illness Duration,y**	18.06 ± 7.49	NA	NA	NA
PANSS				
**PANSS -T**	74.96 ± 17.90	NA	NA	NA
**PANSS-P**	13.60 ± 5.37	NA	NA	NA
**PANSS-N**	23.90 ± 7.56	NA	NA	NA
WCST				
**Categories achieved numbers**	3.24 ± 1.63	6.02 ± 1.65	3.71	<0.001
**Total errors**	39.24 ± 11.56	23.22 ± 6.98	3.72	<0.001
**Perseverative errors**	21.36 ± 9.06	11.23 ± 6.97	3.05	0.026
**Non-perseverative errors**	19.02 ± 6.92	10.98 ± 6.46	3.34	<0.001
**Percentage of conceptual level response**	38.65 ± 18.01	48.92 ± 23.95	−3.96	<0.001

Data were presented as the range of minimum-maximum (mean ± SD). PANSS, the Positive and Negative Syndrome Scale; PANSS-T, the total PANSS score; PANSS-P, the positive score; PANSS-N, the negative score; WCST, the Wisconsin Card Sorting Test; y, year; F, female; M, male. χ ^2^, variance; P<0.05. NA represents missing values or unmeasured data.

### 3.2 Spatial distribution of brain networks between the two groups

**Figure 2:**
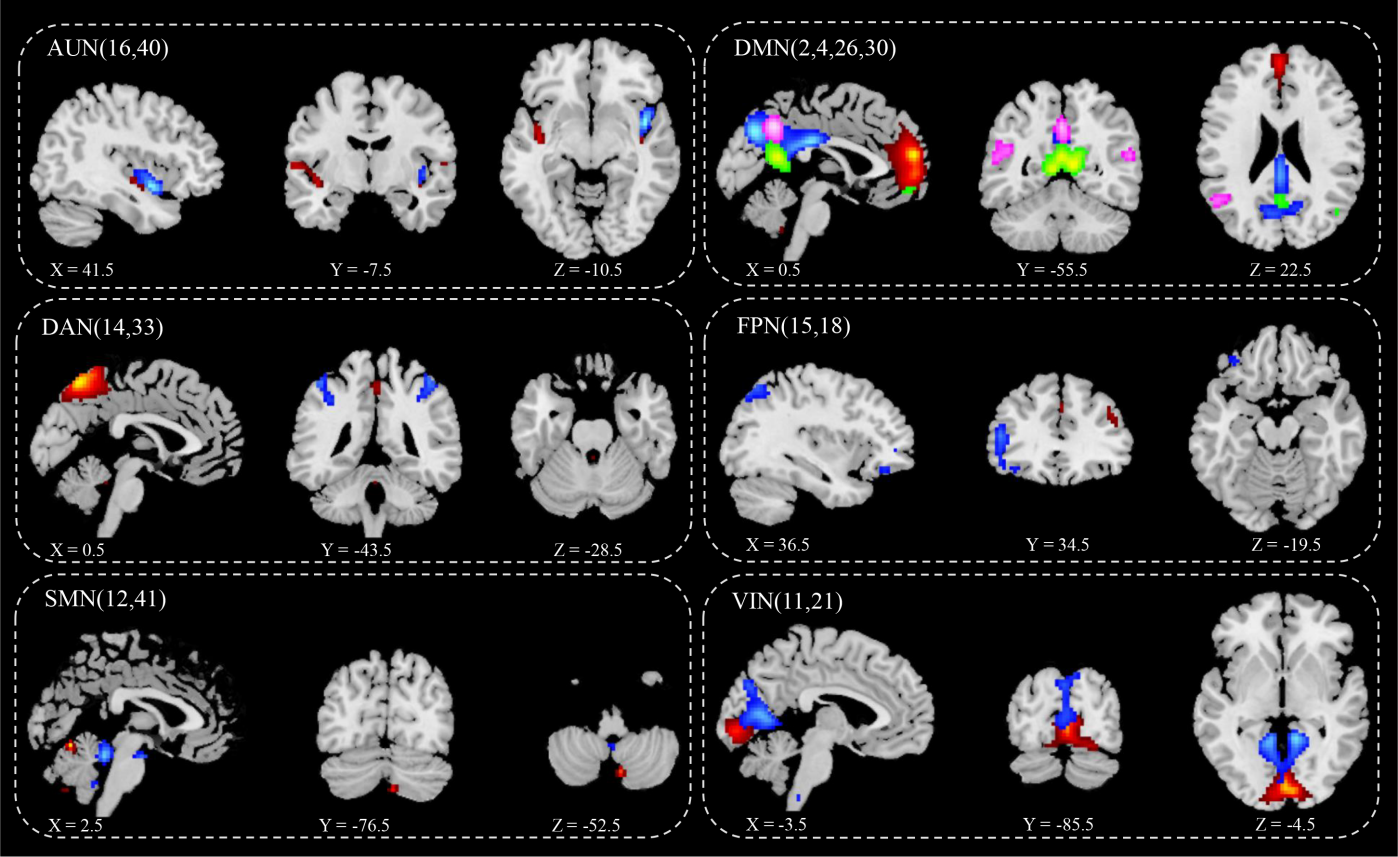
IC spatial divisions of different brain networks. AUN, Auditory Network, blue (16), red (40); DMN, Default Mode Network, blue (2), red (4), green (26), pink (30);DAN, Dorsal Attention Network, blue (14), red (33); FPN, Fronto-parietal Network, blue (15), red (18); SMN, Somatomotor Network, blue (12), red (41); VIN, Visual Network blue (11), red (21); x y z, spatial coordinates. P value < 0.05.

ICA screened out 41 brain network components, of which components 16 and 40 belonged to AUN; components 2, 4, 26, and 30 belonged to DMN; components 33 and 14 belonged to DAN; component 18 belonged to LFPN; component 15 belonged to RFPN; components 12 and 41 belonged to SMN; and components 11 and 21 belonged to VIN. Based on the results for the single-sample t-test group, six brain networks were marked in the two groups (**Figure 2**):

1) AUN: component 16 (Fusiform_L, Temporal_Sup_R, Frontal_Inf_Orb_L, Frontal_Mid_Orb_L, Calcarine_L, Temporal_Sup_L, Calcarine_R, Frontal_Mid_L, Caudate_L, Postcentral_L, Cingulum_Mid_L, Frontal_Sup_R and Thalamus), component 40 (Cerebelum_9, Vermis_9, Temporal_Mid_L, Lingual_R, Thalamus_L, and Temporal_Sup_R);
2) DMN: component 2 (Lingual_L, Vermis_4_5, and Cuneus_L), component 4 (Cingulum_Mid_L), component 26 (Angular_R), component 30 (Rolandic_Oper_R and Frontal_Inf_Tri_L);
3) DAN: component 14 (Cerebelum_9, Pallidum_L, and Insula_R), component 33 (Vermis_6, ParaHippocampal_L, Temporal_Pole_Sup_L, Cerebelum_4_5_L, Thalamus, Temporal_Mid_R, and Cingulum_Mid_R);
4) LFPN: component 18 (Cerebelum_4_5_L, Thalamus, Lingual, Insula, Temporal_Sup_R and Frontal_Mid_L);
5) RFPN: component 15 (Rectus_L Precuneus_R, Calcarine, Cingulum_Post, Temporal_Mid, Frontal_Inf_Orb, Frontal_Med_Orb, Frontal_Inf_Tri, Thalamus, Frontal_Sup_L, Occipital_Mid_L, and Frontal_Mid);
6) SMN: 12 (Cerebelum_6, Thalamus_L, Frontal_Mid_L, Insula_R and Supp_Motor_Area), 41(Cerebelum_4_5, Cerebelum_9);
7) VIN: component11 (Cerebelum_9_L, Cerebelum_7b_L, Frontal_Mid, Cerebelum_4_5_R, Temporal_Mid_L, Frontal_Inf_Orb_L, Temporal_Sup_L, Precuneus_R, Cingulum_Mid_R, and Parietal_Inf_L), component21(Cerebelum_8, Cerebelum_6, Frontal_Mid, ParaHippocampal_L, Temporal_Mid, Occipital_Mid_R and SupraMarginal).

### 3.3 Differences between the brain networks of the two groups

Comparison of the RSN of ICA at the group level revealed significant differences in the FCs within the brain network between the two groups (**Table 2**). Specifically, the significantly different clusters between the two groups were: component 14 (Cerebelum_9_R), component 2 (Lingual_L, Vermis_4_5 and Cuneus_L), component 15 (Rectus_L, Calcarine_L, Cingulum_Post_L, Frontal_Mid_R and Frontal_Sup_L), component18 (Thalamus_L, Thalamus_R) and component 41 (Cerebelum_9_L). The small cluster size of 14 (Cerebelum_9_R) yields low analytical significance; therefore, this region was not included as an ROI.

**Table 2:** Spatial distribution of brain network differential clumps between groups.

Region		Y	X	T	Cluster size (mm^2^)
DAN (component 14)					
**Cerebelum_9_R**	12	−45	−36	4.346	1
DMN (component 2)					
**Lingual_L**	−18	−75	−9	4.37	85
**Vermis_4_5**	6	−45	3	4.841	11
**Cuneus_L**	−6	−90	30	4.269	10
LFPN (component 18)					
**Thalamus_L**	−3	−30	0	4.292	16
**Thalamus_R**	6	−30	0	4.228	17
RFPN (component 15)					
**Rectus_L**	−12	51	−12	4.913	12
**Calcarine_L**	−6	−66	15	3.89	9
**Cingulum_Post_L**	−6	−48	24	4.676	31
**Frontal_Mid_R**	27	36	33	5.361	30
**Frontal_Sup_L**	−24	36	39	4.121	9
SMN (component 41)					
**Cerebelum_9_L**	−3	−51	−54	4.204	10

x y z, spatial coordinates; T, The statistical value of the T-test; F, The statistical value of the F-test.

**Figure 3:**
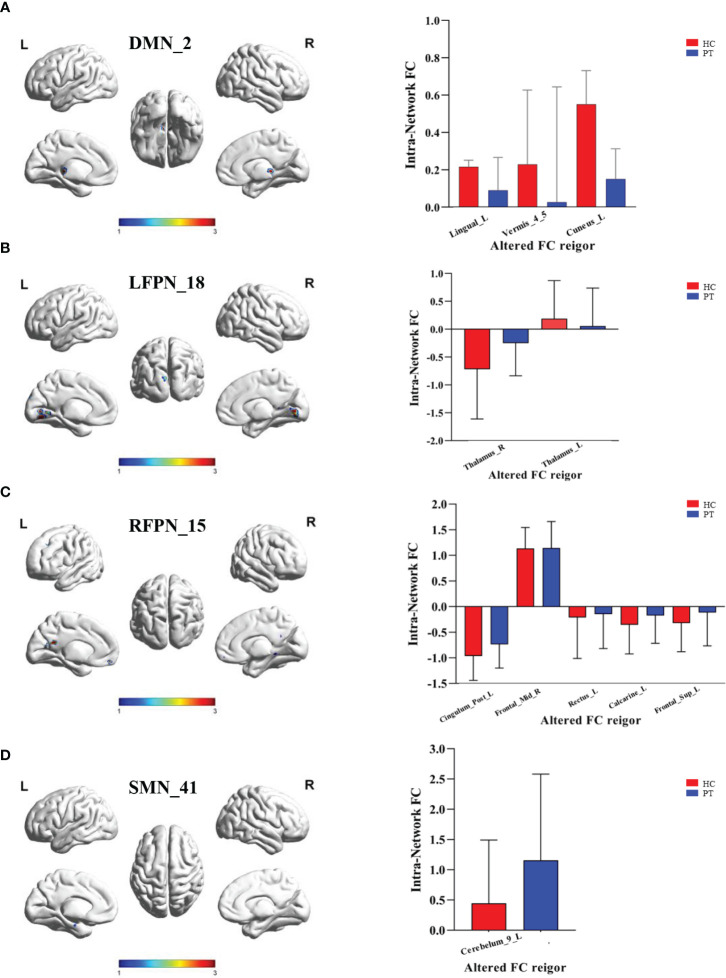
Inter group differences in the ROI of intranetwork FC. **(A)** Inter group differences in the ROI of component 2 of DMN. ROI included Lingual_L, Vermis_4_5 and Cuneus_L; **(B)** Inter group differences in the ROI of component 18 of LFPN. ROI included Thalamus_R and Thalamus_L; **(C)** Inter group differences in the ROI of component 15 of RFPN. ROI included Cingulum_Post_L, Frontal_Mid_R, Rectus_L,Calcarine_L and Frontal_Sup_L; **(D)** Inter group differences in the ROI of component 41 of SMN. ROI included Cerebelum_9_L. DMN, Default Mode Network; LFPN, lift Fronto-parietal Network; RFPN, right Fronto-parietal Network; SMN, Somatomotor Network; L, lift; R, right. P value < 0.05.

Compared with HC group, PT group showed decreased FC in the Lingual_L, Vermis_4_5, and Cuneus_L regions of the DMN (**Figure 3A**). A decrease in the two-sided thalamus FCs of the LFPN is shown in **Figure 3B**. In the RFPN, the FC of Cingulum_Post_L, Rectus_L, Calcarine_L, and Frontal_Sup_L decreased, while that of Frontal_Mid_R did not change significantly (**Figure 3C**). Cerebellum_9_L FC enhancements in SMNs were shown in **Figure 3D**.

### 3.4 Intergroup differences in connectivity between RSN between the two groups

**Figure 4:**
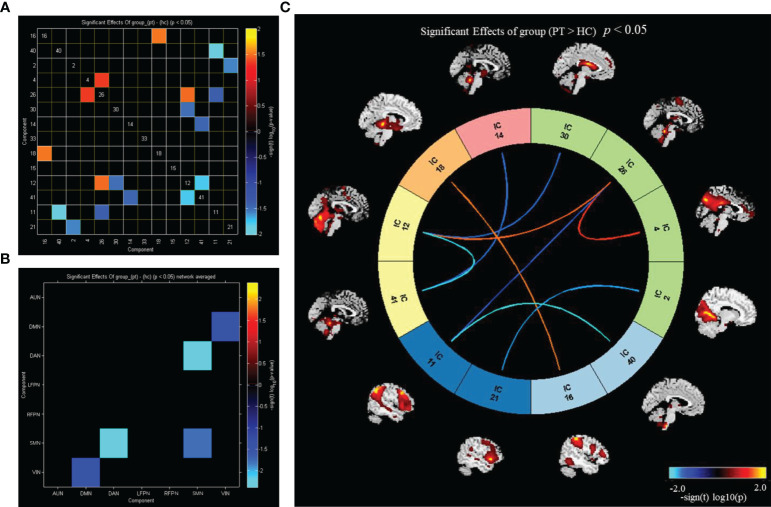
Inter group differences in connectivity between RSNs. **(A)** Inter group differences in connectivity between ICs; **(B)** inter group differences in connectivity between brain networks; **(C)** the ICs connectivity picture of internetwork FC; P value < 0.05.

Utilizing FNC technology, the differences in components between the two groups were analyzed. Compared with HC, in PT, component 16 showed enhanced connection with component 18; component 40 showed reduced connection with component 11; component 2 showed reduced connection with component 21; component 4 showed enhanced connection with component 26; component 26 showed enhanced connection with component 12 and decreased connection with component 11; component 30 showed reduced connection with component 12; component 14 showed reduced connection with component 41; and component 12 showed reduced connection with the component 41 (P<0.05) (**Figure 4A**). Compared with that in HC, the connection between DMN and VIN and between DAN and SMN was weakened in PT (P<0.05) (**Figure 4B**).

Compared with HC, PT exhibited enhanced inter-network connectivity between AUN(16)–LFPN(18), DMN(26)–SMN(12), and DMN(4)–DMN(26), while the inter-network connections between DAN(14)–SMN(41), DMN(30)–SMN(12), DMN(2)–VIN(21), DMN(26)–VIN(11), AUN(40)–VIN(11), and SMN(12)–SMN(41) was reduced (P<0.05) (**Figure 4C**).

### 3.5 Correlation analysis between brain network FC and clinical indicators

**Figure 5:**
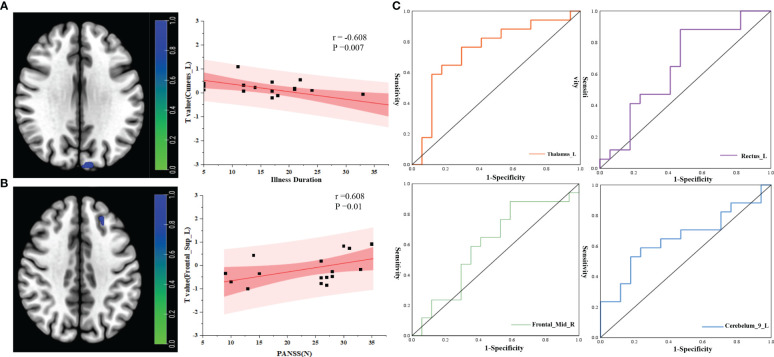
Correlation analysis between RSNs and clinical indicators. **(A)** Correlation analysis between illness duration and ROI; **(B)** correlation analysis between PANSS(N) and ROI; **(C)** ROC curve analysis of the ROIs between the two groups. r, correlation coefficient; p, P value.

Correlation analysis of brain network changes in the ROIs revealed a significant correlation between the FC of the midbrain network and clinical data in PT. In PT, after correcting for confounders, the FC of Cuneus_L was negatively correlated with process (r=−0.608, P=0.007) (**Figure 5A**) and the FC of Frontal_Sup_L was positively correlated with PANSS(N) (r=0.608, P=0.01) (**Figure 5B**).

### 3.6 ROC curve analysis of FC of brain networks in the two groups

ROC curve analysis of the brain network connectivity values based on the ROIs and clinical data of the two groups: when the value of Thalamus_L was −0.196, the sensitivity was 58.8%, the specificity was 88.23%, and the AUC was 0.744 (95% CI: 0.569–0.919); when the value of Rectus_L was −1.106, the sensitivity was 88.2%, the specificity was 52.94%, and the AUC was 0.651 (95% CI: 0.460–0.841); when the value of Frontal_Mid_R was −0.720, the sensitivity was 88.2%, the specificity was 41.17%, and the area under the AUC was 0.599 (95% CI: 0.402–0.795); When the value of Cerebelum_9_L was 0.728,the sensitivity was 58.82%, the specificity was 76.47%, and the area under the AUC was 0.654 (95% CI: 0.465–0.843) (**Figure 5C**).

## 4 Discussion

ICA—a reliable method for analysis of resting-state brain function—enables separation of the mixed signals of intrinsic FCs of whole-brain voxels in time and space, and can obtain independent time series and spatial distribution maps to facilitate analysis of the differences in FCs within and between RSNs (30). In this study, ICA and FNC techniques were used to explore the changes in brain network FC in patients with chronic schizophrenia. Compared with that of HC, the FC of the left lingual lobe, cuneiform lobe, and cerebellar vermis region in the DMN was found to be lower in PT. Furthermore, we observed decreased FC in the bilateral thalamic regions as well as in the left cingulate gyrus and right frontal lobe. In the SMN, FC in the left cerebellar region was observed to be reduced. Correlation analysis of brain network changes in the ROIs further revealed a significant correlation between the changes in brain network FC and clinical data.

### 4.1 Patients with chronic schizophrenia have abnormal connections within the brain network

Schizophrenia is a mental disorder caused by brain dysfunction, and patients may exhibit abnormal function of multiple brain regions (31). The DMN, an important endogenous network that enhances activity when the brain is at rest, plays an important role in self-referential thinking and reflection as well as in maintaining the brain’s internal activity during the resting state. The DMN functional areas include the medial prefrontal cortex, the posterior cingulate cortex, the inferior parietal cortex, and the anterior cuneus (32). These brain regions are involved in a variety of higher-order cognitive functions such as memory, anticipation, and self-processing (33, 34). The results of our study show a decrease in the FC of the Lingual_L, Vermis_4_5, and Cuneus_L regions of the DMN, which is consistent with the clinical characteristics of patients with chronic schizophrenia. In FPN and DAN, which are both high-order networks, we find that a decrease in the two-sided thalamus functional connections in the LFPN as well as in the Cingulum_Post_L, Rectus_L, Calcarine_L, and Frontal_Sup_L functional connections in the RFPN. These brain networks play an important role in cognitive function, and the decline in their FC is consistent with the clinical manifestations of cognitive impairment in patients with chronic schizophrenia. The study find that the thalamus as the most prominent biomarkers out of many towards the diagnosis of schizophrenia and HC.In our study, PT were schizophrenia patients with long disease course and repeated episodes, which should theoretically have strong biological characteristics. In comparison with HC, the functional connection of the thalamus was abnormal, which should also prove the above research (9).

Studies have shown that schizophrenia is not only associated with impaired higher-order cognitive functions, but also deficits in multisensory integration and perceptual processing (35). The AUN of the brain is located in the bilateral temporal cortex, and its main role is to process and process auditory information. A functional magnetic resonance meta-analysis of auditory hallucinations demonstrated that these hallucinations were associated with the medial temporal lobe, which is involved in auditory verbal memory (36). In our study, no significant decline in FC of the AUN was found, which may be related to the long duration of the disease, mostly hypofunctional manifestations, and the lack of obvious positive symptoms. The human brain has a cortical stratification mechanism that extends from the primary sensory system to the higher cognitive function system, which helps the human brain process different domains of functions (such as sensory and cognitive processes) separately. In addition, the brain network can be dynamically configured and interacted to achieve more complex mental activities (37).

Patients with schizophrenia exhibit disorders in sensory processing and integration of higher-order cognitive functions (38). Disordered interaction between higher-order cognitive functions and perceptual processing deficits play an important role in the pathogenesis of schizophrenia (39). Abnormalities in the FC between the sensorimotor system and the higher-order cognitive system may also be associated with mutual inhibition, disruption of the patient’s neurobiological circuitry, and disturbance of the balance between excitation and inhibition (40, 41). In our study, we found that patients with chronic schizophrenia had weakened connectivity between the DMN and the VIN, and the DAN exhibited weakened connectivity with the SMN. These findings verify the core defects of schizophrenia as supported by the “disconnection hypothesis” (42).

### 4.2 Correlation between brain network FC and clinical manifestations in patients with chronic schizophrenia

Correlation analysis of brain network changes in the ROIs revealed that there was a significant correlation between the changes in brain network FC and clinical data in PT. The FC of the left cuneiform lobe in the default network of PT was negatively correlated with the course Cuneus_L of the disease—i.e., the longer the course of the disease, the weaker the connectivity, indicating that the longer disease course resulted in disruption of the DMN connection of brain function. In our previous studies, we found that patients with chronic schizophrenia had more prominent cognitive impairment (19). In this study, we found that Frontal_Sup_L FC was positively correlated with PANSS(N) (r=0.608, P=0.01) (**Figure 4B**); furthermore, we discovered that PT had more prominent negative symptoms and a greater difference in Frontal_Sup_L FC compared with HC. This result is consistent with those of previous studies. In our study, the AUC for the differences in the cuneiform lobe, thalamus, and Wisconsin classification test scores were higher, showing that these indicators have higher sensitivity for the diagnosis of chronic schizophrenia and provide a potential direction for future research.

## 5 Limitations and challenges

There are some limitations in this study, firstly, this study is a single-center study, with certain regional and selective bias, and the sample size is relatively small, which may reduce the value of the study results. Secondly, This results in reduced levels of test–retest reliability of rs-fMRI measures in studies utilizing repeated-measures designs in which the participants are scanned multiple times over a specific timeframe. Variabilities in the resting-state signal within individuals are also region-specific, with brain regions involved in working memory, inhibition, attention, and language showing higher levels of intra-individual variabilities than that of the somatomotor or auditory network (4). Another point of concern is the high dimensionality of rs-fMRI data, in which methods such as ICA or principal component analysis are used to reduce the dimensionality by parcellating the whole brain into smaller areas, but the optimal number of brain units to be used is still not clear (43).

## 6 Conclusion

The changes of Structural and functional connectivity of specific brain regions in patients with chronic schizophrenia may be related to a long illness duration chronic schizophrenia (10 years or more). This provides a theoretical basis for the early diagnosis and clinical prognosis evaluation of a long illness duration chronic schizophrenia.

## Data availability statement

The original contributions presented in the study are included in the article/supplementary material. Further inquiries can be directed to the corresponding authors.

## Ethics statement

The studies involving humans were approved by Ethics Committee Mental Health Center of Inner Mongolia Autonomous Region. The studies were conducted in accordance with the local legislation and institutional requirements. The participants provided their written informed consent to participate in this study. Written informed consent was obtained from the individual(s) for the publication of any potentially identifiable images or data included in this article.

## Author contributions

YB: Conceptualization, Supervision, Writing – review & editing. JXL: Data curation, Formal analysis, Methodology, Software, Validation, Visualization, Writing – original draft, Writing – review & editing. DP: Formal analysis, Resources, Writing – original draft. JS: Data curation, Resources, Writing – original draft. YG: Resources, Visualization, Writing – original draft. LH: Formal analysis, Investigation, Writing – original draft. BT: Formal analysis, Methodology, Writing – original draft. XH: Conceptualization, Supervision, Writing – original draft. JYL: Data curation, Software, Visualization, Writing – original draft. ZL: Conceptualization, Supervision, Writing – original draft. FY: Methodology, Project administration, Resources, Writing – review & editing.
